# Oxygen‐free Layer‐by‐Layer Assembly of Lithiated Composites on Graphene for Advanced Hydrogen Storage

**DOI:** 10.1002/advs.201600257

**Published:** 2017-04-25

**Authors:** Guanglin Xia, Yingbin Tan, Xiaowei Chen, Fang Fang, Dalin Sun, Xingguo Li, Zaiping Guo, Xuebin Yu

**Affiliations:** ^1^ Department of Materials Science Fudan University Shanghai 200433 China; ^2^ Institute for Superconducting and Electronic Materials University of Wollongong North Wollongong NSW 2522 Australia; ^3^ College of Chemistry and Molecular Engineering Peking University Beijing 100871 China

**Keywords:** complex hydrides, graphene, hydrogen storage, layer‐by‐layer, lithiation

## Abstract

**A facile hydrogenation‐induced self‐assembly strategy** to synthesize lithium hydride (LiH) nanosheets with a thickness of 2 nm that are uniformly distributed on graphene is reported and designed. Taking advantage of LiH nanosheets with high reactivity and a homogeneous distribution on graphene support as a nanoreactor, the confined chemical synthesis of oxygen‐free lithiated composites is effectively and efficiently realized.

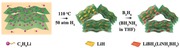

Nanostructured oxygen‐free lithiated composites (LiH, LiNH_2_BH_3_, LiBH_4_, Li_3_N, etc.) have received great attention in the field of hydrogen storage due to their high hydrogen content.^1^, ^2^ For example, LiBH_4_ possesses a high gravimetric (18.5 wt%) and volumetric (121 kg m^−3^) hydrogen density, which has been widely regarded as a promising candidate for onboard applications.^2^, ^3^ The high themodynamic stability, sluggish dehydrogenation/hydrogenation kinetics, and poor reversibility, however, restrict their practical applications as hydrogen storage materials.^4^ The reduction of particle size down to the nanometer scale could effectively shorten diffusion paths for hydrogen and mass transport, increase the surface‐to‐volume ratios of particles, and improve the interfacial reactivity during reversible hydrogen storage reactions, leading to significantly improved hydrogen storage performance.^5^ Among them, lithium metal and/or its hydrides are the major constituents, which could be directly used as precursors for oxygen‐free lithiation of various composites, but their chemical synthesis still remains a big challenge owing to their high reactivity and incompatibility with all conventional ligands, and it becomes the more challenging, the smaller the particles are.^6^ Moreover, nanostructured particles are intrinsically unstable and tend to grow into larger crystallites, which would result in continuous degradation of the nanostructure‐induced improvement of hydrogen storage performance.^7^ Hence, it will be of great importance and necessity to develop a robust nanostructure of lithiated composites for delivering outstanding and durable hydrogen storage performance.

One alternative method to synthesize and stabilize nanostructured materials is adopting templates to support their nucleation and growth during the self‐assembly process, which could further prevent the agglomeration and sintering of nanosized materials during practical applications.^8^, ^9^, ^10^ Graphene, which is a single layer of carbon atoms arranged in a honeycomb structure, has proved to be an exceptionally promising and versatile building block for anchoring diverse nanostructured materials due to its fascinating electronic, mechanical, and thermal properties, high specific surface area, light weight, and flexibility.^11^ In fact, due to its capability for electrochemical reactions with lithium, the interaction of lithium metal in layer‐structured graphite has been intensively investigated, leading to the formation of Li*_x_*C_6_ (0 < *x* ≤ 1). This method is limited, however, by the low and inhomogeneous loading of lithium in the composite, i.e., a loading capacity of 8.8 wt% for LiC_6_, which refers to the composition with maximal intercalation. The low loading‐concentration of nanostructured active materials would lead to significantly limited energy density of the whole composite, e.g., low hydrogen storage capacity as hydrogen storage material.^12^ Moreover, possible contamination by the electrolyte is difficult to avoid, and particle agglomeration and distribution are often hard to control during this procedure.^13^ Therefore, the development of a general and facile strategy to synthesize nanostructured lithium metal and/or its hydrides with homogeneous distribution and outstanding stability is highly desirable and, indeed, imperative for advanced applications as hydrogen storage materials.

Herein, we report a facile and scalable wet‐chemical synthesis of LiH nanosheets with a thickness of only 2 nm, uniformly distributed on graphene through the hydrogenation of butyllithium under high pressure. The formation of LiH nanosheets not only stabilizes the high reactivity of lithium metal during the synthetic procedure, but also provides a diverse precursor that can be used to fabricate various oxygen‐free lithiated composites. Due to the favorable adsorption of butyllithium on graphene, the selective and favorable growth of LiH nanosheets on graphene could be achieved. The porous structure, uniform distribution, and robust and flexible graphene sheets in the as‐prepared LiH@graphene hybrid (denoted as LiH@G) give it the properties of an ideal nanoreactor for oxygen‐free lithiation of diverse composites to fabricate lithiated composites. As a proof of concept, by adopting LiH@G as a nanoreactor, we demonstrate a solid–liquid reaction and a solid–gas reaction to synthesize LiNH_2_BH_3_ and LiBH_4_ layers with a thickness of ≈4 nm, respectively, which present a layer‐by‐layer nanostructure, inheriting the structure of the LiH nanosheets homogeneously anchored on the graphene (**Figure** **1**). It is demonstrated that this technique is general enough for oxygen‐free layer‐by‐layer assembly of various lithiated compoites. The bottom‐up self‐assembly process coupled with the high surface area of graphene leads to a high loading of LiH and hence, LiNH_2_BH_3_ and LiBH_4_ nanolayers with a homogeneous distribution, resulting in high system hydrogen storage capacities and energy density. Moreover, the synergistic effects of nanosizing, the convenient channels for fast transportation of hydrogen, the high thermal conductivity, and the catalytic effect of graphene as the structural support contribute to the significantly improved kinetics of hydrogen release and uptake. Furthermore, the growth and agglomeration of nanoparticles and the stress caused by the large volume changes during continuous cycles of hydrogenation and dehydrogenation could be significantly alleviated by the layer‐by‐layer nanostructure, leading to excellent cycling stability. When evaluated as hydrogen storage materials, all the synthesized lithiated materials exhibit significantly enhanced performance with stable cycling. This approach provides a new perspective for the oxygen‐free layer‐by‐layer assembly of lithiated composites toward advanced performance.

**Figure 1:**
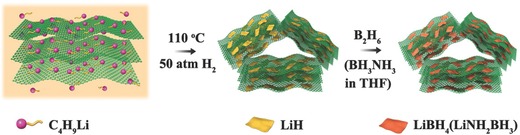
Schematic illustration of the fabrication of LiH@G, which was then adopted as a nanoreactor for oxygen‐free layer‐by‐layer assembly of LiBH_4_@G and LiNH_2_BH_3_@G through a solid–gas reaction and a solid–liquid reaction, respectively.

As illustrated in Figure 1, LiH@G was first synthesized by a one‐step solvothermal reaction of butyllithium supported by graphene in cyclohexane under a hydrogen pressure of 50 atm. During this process, LiH was formed upon the hydrogenation of butyllithium in situ anchored on graphene, and the X‐ray diffraction (XRD) pattern (Figure S1 in the Supporting Information) of the as‐prepared LiH@G verifies the formation of LiH after solvothermal treatment. Scanning electron microscopy (SEM) and transmission electron microscopy (TEM) disclosed the selective growth of 2D LiH nanosheets on graphene, which appear to be transparent (**Figure** **2**a–d), with a typical thickness of ≈2 nm and a lateral size ranging from 50 to 150 nm. The interplanar spacing of the particle lattice revealed by the high‐resolution TEM (HRTEM) image (Figure 2e) is ≈0.203 nm, which agrees well with the (200) lattice spacing of LiH and is consistent with the XRD results. Further evidence for the formation of LiH after solvothermal treatment is provided by energy‐dispersive X‐ray spectroscopy (EDX; Figure S2, Supporting Information), X‐ray photoelectron spectroscopy (XPS; Figure S3, Supporting Information), and Fourier‐transform infrared (FTIR; Figure S4, Supporting Information) spectroscopy. No agglomeration of LiH nanosheets was observed in the LiH@G, while pure LiH synthesized under the same experimental conditions without the presence of graphene tends to aggregate with serious stacking owing to the high surface energy (Figure 2f). These results confirm that the presence of graphene plays an important role in the generation of highly dispersed and homogeneous LiH nanosheets.

**Figure 2:**
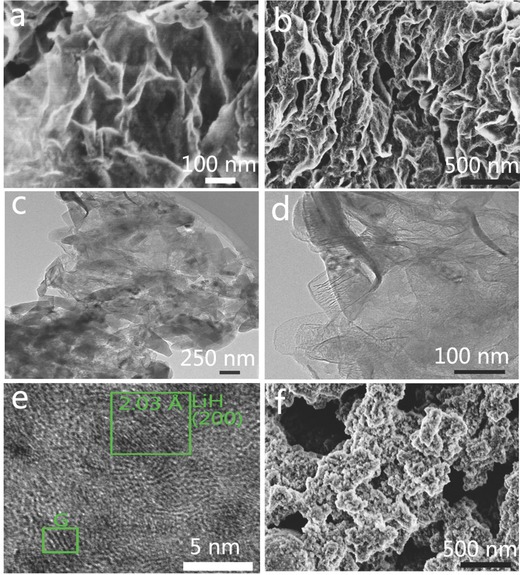
a,b) SEM images, c,d) TEM images, and e) HRTEM image of LiH@G. f) SEM image of LiH synthesized under the same conditions without the presence of graphene.

Since the adsorption of precursors on graphene plays an important role in the self‐assembly of nanostructured materials on graphene^9^ and only C_4_H_9_Li and C_6_H_12_ are involved in the synthesis of LiH@G, density‐functional theory (DFT) calculations were conducted (**Figure** **3**) to investigate the competitive adsorption of C_4_H_9_Li and C_6_H_12_ on graphene in order to unravel the mechanism for the growth of LiH nanosheets on graphene. It confirms that the binding energy between C_4_H_9_Li and graphene is −0.616 eV, with an equilibrium distance of 2.57 Å under the most stable adsorption configuration (Figure 3a–d), which is much lower than that of the C_6_H_12_‐Graphene system, i.e., −0.309 eV for an equilibrium distance of 2.52 Å. Therefore, the driving force for the growth of LiH nanosheets on graphene could be attributed to the favorable adsorption of C_4_H_9_Li on graphene compared to C_6_H_12_.^14^ Prompted by this unique nanoarchitecture, it is expected that the as‐prepared LiH@G can act as a universal nanoreactor to fabricate various oxygen‐free lithiated composites. In particular, the sheet‐on‐sheet structure of LiH@G could effectively suppress the agglomeration of LiH nanosheets and alleviate particle growth and aggregation during chemical reactions, thus protecting the structural integrity. Moreover, the large out‐of‐plane macropores, ranging from tens of nanometers to a few micrometers, which are afforded by the assembly of graphene sheets, could be clearly observed from the cross‐sectional view (Figure 2b). Both out‐of‐plane macropores and in‐plane nanopores originating from the cross‐linking of LiH nanosheets and flexible graphene nanosheets (Figure 2a and Figure S5, Supporting Information) provide facile pathways for the transport of reactants and buffer the volume changes during chemical transformation, which is beneficial in regard to alleviate the growth and agglomeration of nanoparticles. Furthermore, the ultrathin LiH nanosheets could facilitate the diffusion of ions and mass transport to promote enhanced chemical transformation. As a proof of concept, a solid–gas reaction to synthesize LiBH_4_ and a solid–liquid reaction to synthesize LiNH_2_BH_3_ were attempted by adopting LiH@G as the nanoreactor.

**Figure 3:**
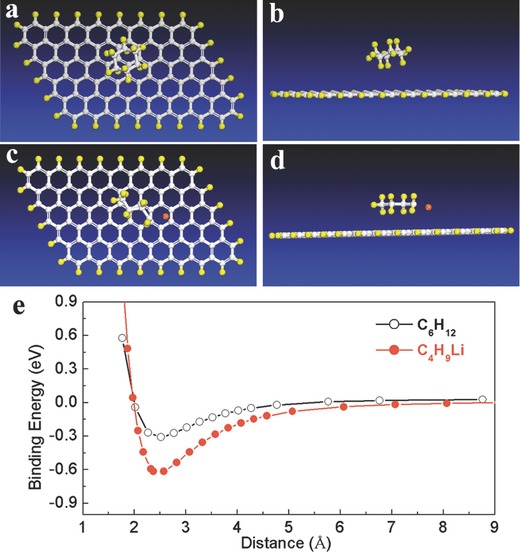
Atomic structures for the adsorption of C_6_H_12_ (a) top and b) side views) and C_4_H_9_Li (c) top and d) side views) on graphene in the most stable configuration. e) Binding energy curves for adsorption of C_6_H_12_ and C_4_H_9_Li on the graphene, based on DFT calculations.

After the chemical reaction of LiH@G with B_2_H_6_ and BH_3_NH_3_, the XRD patterns (Figure S6, Supporting Information) and the FTIR results confirm the formation of LiBH_4_ (Figures S7, Supporting Information) and LiNH_2_BH_3_ (Figure S8, Supporting Information) with high purity, respectively.^15^ Taking advantage of the mechanical stiffness of graphene, the chemical transformation based on the solid–gas/solution reaction could be effectively supported, which ensures that the nanostructure could be inherited from the corresponding LiH nanosheets and simultaneously avoids severe agglomeration of the freshly formed products. Consequently, the as‐formed LiBH_4_/graphene hybrids (LiBH_4_@G) and LiNH_2_BH_3_/graphene hybrids (LiNH_2_BH_3_@G) exhibit a curved thin, flaky morphology, in which LiBH_4_ and LiNH_2_BH_3_ nanosheets are uniformly anchored on the graphene (**Figure** **4**a,e and Figure S9, Supporting Information), leading to the assembly of a layer‐by‐layer nanostructure. TEM images (Figure 4b,c,f) clearly exhibit typical layered platelet‐like morphology, homogeneously distributed on graphene, and demonstrate that the resultant hybrid well preserves the 2D nanosheet structure with no obvious agglomeration, validating the important role of graphene in stabilizing the as‐synthesized nanostructure. The 3D size of the as‐formed LiBH_4_ and LiNH_2_BH_3_ is slightly larger than that for the relevant LiH nanosheets, owing to the large‐scale incorporation of foreign elements. HRTEM images of the curled edge show the heterointerfaces between LiBH_4_ nanolayers and graphene (Figure 4d), which indicate that the thickness of the as‐formed LiBH_4_ nanolayer is ≈4 nm, and there are lattice fringes with an interplanar spacing of 0.305 nm (inset of Figure 4d), which could be indexed to the (201) lattice planes of LiBH_4_ and agree well with the XRD results (Figure S6, Supporting Information). No free LiBH_4_ and LiNH_2_BH_3_ nanosheets could be observed, and all of them remain uniformly anchored on graphene, even after long‐term ultrasonic dispersion for TEM characterization, which suggests the relatively strong interfacial interaction between LiBH_4_ or LiNH_2_BH_3_ and graphene. This therefore verifies the great potential of LiH@G as a smart nanoreactor for the oxygen‐free layer‐by‐layer assembly of lithiated composites toward enhanced performance in various fields.

**Figure 4:**
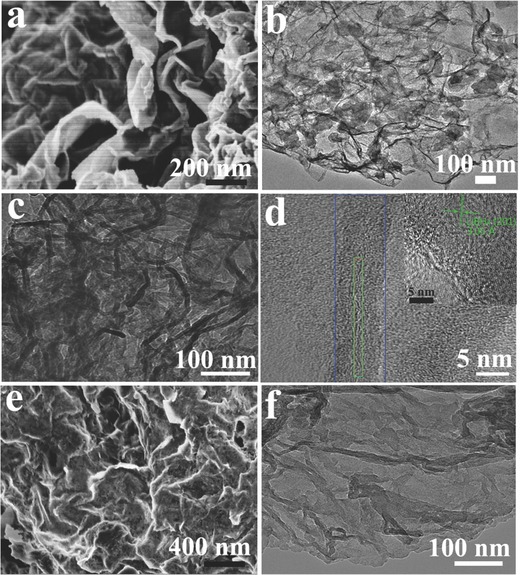
SEM images of the as‐synthesized a) LiBH_4_@G and e) LiNH_2_BH_3_@G. TEM images of b,c,d) LiBH_4_@G and f) LiNH_2_BH_3_@G. The inset of (d) is an HRTEM image of LiBH_4_@G.

When evaluated as hydrogen storage materials, the introduction of B_2_H_6_ and NH_3_BH_3_ with high H_2_ capacity in LiH@G significantly upgrades the loading ratio of the as‐synthesized products and thereby improves the energy density in addition to the structural stability, e.g., a practical H_2_ capacity of 6.8 wt%, corresponding to ≈50 wt% for LiH in the composite, which could be increased to a practical H_2_ capacity of 12.8 wt%, corresponding to ≈69.1 wt% for LiBH_4_ in the composite. Simultaneously, the uniform distribution of LiH nanosheets and the steric confinement of graphene as structural support preserve the structural integrity and the nanosize effects of the as‐synthesized products. This unique sandwich configuration coupled with nanosize effects leads to significantly improved hydrogen storage performance based on a series of measurements. The mass spectra (MS) indicate that both the onset temperature (*T*_onset_) and the peak temperature (*T*_peak_) are considerably reduced for LiH, LiNH_2_BH_3_, and LiBH_4_ anchored on graphene as compared to their bulk counterparts (Figure 3a and Figure S10, Supporting Information). Specifically, LiH@G exhibits a *T*_peak_ at 445 °C, and complete dehydrogenation could be accomplished at ≈500 °C with a weight loss of ≈6.8 wt%, while no hydrogen was released from the bulk LiH, even when it was heated up to ≈550 °C (**Figure** **5**a). Additionally, LiNH_2_BH_3_@G presents a *T*_onset_ of 53 °C, 15 °C lower than for bulk LiNH_2_BH_3_ (Figure S10, Supporting Information), with a peak temperature at ≈79 °C. Furthermore, a *T*_peak_ of 346 °C was observed for LiBH_4_@G, 124 °C lower than that of its bulk counterpart, which achieves a practical hydrogen density of 12.8 wt% in the composite (Figure 5b), corresponding to a very high theoretical loading capacity of 69.1% (considering the complete decomposition of LiBH_4_ on the basis of XRD results of the dehydrogenated products (Figure S11, Supporting Information)). By comparison, the loading capacity of LiBH_4_ in templates is usually reported to be as low as 30 wt% or even less.^16^ Moreover, no measureable desorption of diborane was detected for the dehydrogenation of LiBH_4_@G, owing to the tremendous reduction of particle size down to the nanometer scale.^17^, ^18^ It should be noted that only hydrogen was detected from the thermal desorption of the graphene‐supported samples (Figures S12 and S13, Supporting Information), indicating the high purity of the released H_2_. Since LiBH_4_@G hybrid displays the highest hydrogen capacity and favorable thermodynamics toward reversible hydrogen storage performance, LiBH_4_@G was further investigated in detail.

**Figure 5:**
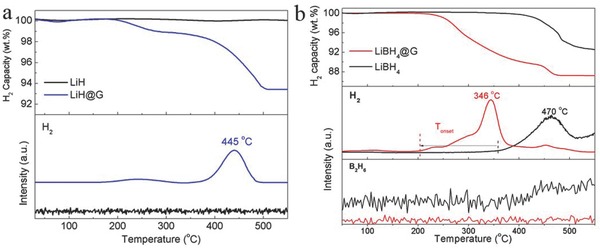
TG (top) and MS (bottom) results for the decomposition of the a) as‐prepared LiH@G and b) LiBH_4_@G in comparison with their bulk counterparts. The hydrogen capacity is calculated on the basis of the whole composite.

The isothermal dehydriding kinetics of LiBH_4_@G were further investigated in comparison with the ball‐milled LiBH_4_ and the composite of LiBH_4_ and graphene (denoted as LiBH_4_/G). It could be clearly observed that only a capacity of less than 5 wt% hydrogen was released from the ball‐milled LiBH_4_ when heated at a temperature as high as 320 °C over a period of 300 min. Due to the catalytic effects of graphene on the hydrogen storage process in LiBH_4_,^19^ LiBH_4_/G with particle sizes of most materials larger than 400 nm and serious aggregation (Figure S14, Supporting Information) exhibited improved dehydrogenation kinetics. Only 5.5 wt% hydrogen, however, could be released from LiBH_4_/G under the same conditions. By comparison, graphene‐supported LiBH_4_ nanolayers exhibited a fast dehydrogenation at only 280 °C with a capacity of ≈7.6 wt%, and the value further increased to ≈ 8.4 wt% and 9.5 wt% at the temperature of 300 and 320 °C, respectively. In particular, 9.7 wt% hydrogen could be rapidly released from LiBH_4_@G at a temperature as low as 340 °C in only 60 min. In order to gain better insight into the improvement of the dehydrogenation kinetics of LiBH_4_ nanolayers, the apparent activation energies (*E*_a_) were quantitatively calculated based on the Arrhenius equation by fitting the experimental dehydriding kinetics at different temperatures (Figure S15, Supporting Information). According to the slope of the linear plot of ln (dehydriding rate) versus 1/*T* (*T*, absolute temperature), the *E*_a_ value for the dehydrogenation from LiBH_4_@G was determined to be 119.6 kJ mol^−1^, which is significantly lower than that reported for its bulk counterpart (189.4 kJ mol^−1^ for the dehydrogenation^18^), which is attributed to the tremendous reduction of particle size and the homogeneous distribution on graphene.

The cycling stability is a crucial parameter for the application of hydrogen storage materials, but the cycling life of LiBH_4_ is significantly limited because the sintering leads to the continuous deterioration of hydrogen storage performance, and the phase separation and aggregation of LiH and B during dehydrogenation results in a significant loss of reversibility.^20^ It could be clearly observed that the H_2_ capacity released from LiBH_4_/G was significantly decreased from 6.3 wt% down to only 2.9 wt% after only three cycles, with a capacity retention of 45.5% for the third cycle. By comparison, the H_2_ capacity still reached 7.5 wt% for graphene‐supported LiBH_4_ nanolayers under the same conditions during the fifth cycle of dehydrogenation, which is a record high reversible storage capacity at the lowest temperature (320 °C), corresponding to a capacity retention of ≈80%. The remarkable reversibility of LiBH_4_@G was further confirmed by the FTIR (Figure S16, Supporting Information) and XRD (Figure S17, Supporting Information) results, which demonstrates the regeneration of LiBH_4_ to its initial state. In addition, the formation of the stable compound Li_2_B_12_H_12_ in the rehydrogenated products was suppressed, which effectively prevents the loss of B during cyclic H_2_ release and uptake, and favors the stable cycling of LiBH_4_.^21^ This phenomenon is also observed in the dehydrogenation process of graphene‐encapsulated NaBH_4_ nanoparticles, which is attributed to the reduction of particle size down to nanometer range.^10^ The SEM, TEM, and scanning TEM (STEM) images confirm that the morphology of LiBH_4_@G is well preserved (Figure 5c) and no obvious agglomeration is found (Figure 5d,e), which validates the high stability of LiBH_4_ nanolayers anchored on graphene, resulting from the intimate physical contact and homogeneous distribution between the graphene and the LiBH_4_, and is responsible for the long‐term cycling stability that was achieved.

In view of the aforementioned considerations, the advanced hydrogen storage performance of LiBH_4_@G could be attributed to the following reasons. First, the nanostructure engineering strategy, based on the adoption of LiH@G fabricated via a bottom‐up self‐assembly process as a nanoreactor, could realize the synthesis of homogeneous LiBH_4_ nanolayers that were uniformly anchored on graphene with a thickness of ≈4 nm and a high loading amount, thus delivering high system energy density. Second, the reduction of particle size down to the nanometer range, leading to shortened diffusion paths and an increased number of grain boundaries, could significantly improve the H‐exchange kinetics for pulling H_2_ into and out of the LiBH_4_ nanolayers (**Figures** 5 and **6**). Moreover, the suppression of diborane (Figure 5) and stable “sinks” e.g., Li_2_B_12_H_12_ (Figure S16, Supporting Information), during the dehydrogenation of LiBH_4_, owing to the reduction of particle size down to ≈4 nm, could circumvent the loss of B during reversible hydrogen storage. Furthermore, in the sandwich‐structured layer‐by‐layer structure between LiBH_4_ and graphene with its high surface area, graphene could act as a space barrier to effectively alleviate the particle growth and sintering of LiBH_4_ to a large extent during the thermal treatment involved in cycling (Figures 3 and 6). Additionally, the homogeneous dispersion of LiBH_4_ nanolayers on graphene with intimate contact could facilitate the catalytic effects of graphene on the hydrogenation and dehydrogenation process of LiBH_4_ and the heat transfer in the composite, leading to further improvement of the hydrogen storage performance of LiBH_4_. Because of the synergistic effects of the aforementioned factors, the LiBH_4_@G exhibits significantly advanced hydrogen storage performance.

**Figure 6:**
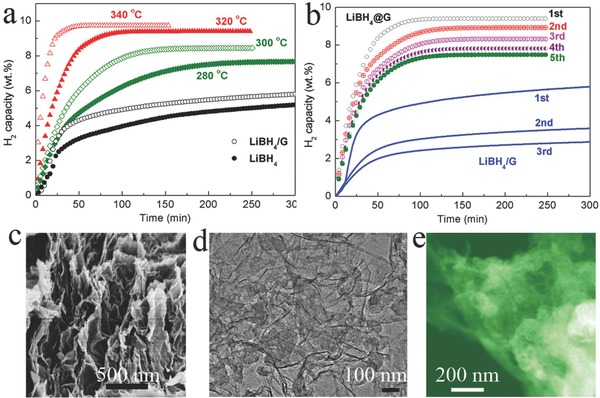
a) Isothermal dehydrogenation of the as‐prepared LiBH_4_@G at various temperatures, with ball‐milled LiBH_4_ and ball‐milled LiBH_4_/G at 320 °C included for comparison. b) Reversible dehydrogenation of hydrogen storage capacities of LiBH_4_@G in comparison with LiBH_4_/G at 320 °C. c) SEM, d) TEM, and e) STEM images of LiBH_4_@G after five cycles of hydrogenation and dehydrogenation. The hydrogen capacity is calculated on the basis of the whole composite.

In summary, we have developed a facile strategy for efficient preparation of graphene‐supported ultrafine LiH nanosheets with both homogeneous distribution and uniform size. Taking advantage of the unique sheet‐on‐sheet architecture, the flexible and robust structure of graphene, and the high reactivity of LiH nanosheets as a smart nanoreactor, a solid–gas reaction and solid–liquid reaction were used to synthesize LiBH_4_ and LiNH_2_BH_3_ nanolayers, respectively, as representative examples. The distinctive features of the resulting graphene‐supported Li‐containing hydrides, with nanocrystallization, homogeneous dispersion, and flexible and porous structure, could not only effectively prevent the growth and aggregation of nanostructured particles to a large extent during the cycling process, facilitate the catalytic effects of graphene on the hydrogenation and dehydrogenation process, and improve the heat transfer in the composite, but also allow for the fast diffusion of mass and hydrogen. As a consequence, the as‐prepared LiBH_4_@G displayed outstanding hydrogen storage performance with respect to the kinetics, thermodynamics, and long‐term cyclability. In addition, we believe that this concept may be also a viable route for the oxygen‐free layer‐by‐layer assembly of various lithiated composites with homogeneous distribution on graphene and could lead to rapid advancement in the development of their promising applications in different areas.

## Experimental Section

*Synthesis of LiH@G*: Graphene‐supported LiH nanosheets (LiH@G) were fabricated via the hydrogenation of *n*‐butyllithium (*n*‐BuLi) in cyclohexane. In a typical synthesis, 2 mL *n*‐BuLi solution (2 m in cyclohexane) and 0.03 g graphene were dispersed in 40 mL cyclohexane in a pressure reactor vessel, and then the reaction was conducted under 50 atm H_2_ at 100 °C for 24 h with the aid of ultrasonication and stirring. After consecutive washing and centrifugation, the resulting composite was dried at room temperature under dynamic vacuum on a Schlenk line, denoted as LiH@G.

*Synthesis of LiBH_4_@G*: As shown in Figure S18 (Supporting Information), LiBH_4_@G hybrids were synthesized by thermal treatment of 0.03 g LiH@G in a mixed B_2_H_6_/H_2_ atmosphere at 120 °C for 3 d. First, the reactor was loaded with LiH@G and a ball‐milled mixture of 0.6 g of ZnCl_2_ and LiBH_4_ with a molar ratio of 1:2, respectively, in the glove‐box. Subsequently, the mixed B_2_H_6_/H_2_ atmosphere was obtained from the decomposition (>90 °C) of a ball‐milled mixture of ZnCl_2_ and LiBH_4_. After the reaction of LiH@G with B_2_H_6_, the reactor was completely degassed via dynamic vacuum for 10 h at 80 °C to obtain LiBH_4_@G.

*Synthesis of LiNH_2_BH_3_@G*: 0.04 g LiH@G was used as the nanoreactor, which was first dispersed in 5 mL tetrahydrofuran (THF) with the aid of sonication, followed by the slow addition of ammonia‐borane (0.0776 g) solution in THF (5 mL). Afterward, the mixture was stirred at room temperature for 3 h. Finally, LiNH_2_BH_3_@G hybrids were obtained by removing the solvent under dynamic vacuum.

*Preparation of LiBH_4_/G Composite*: The bulk LiBH_4_/G composite with a weight ratio of 3:1 was prepared by ball‐milling technique using a planetary QM‐1SP2. The milling procedure with a ball‐to‐powder ratio of 50:1 and a milling speed of 350 rpm was carried out by alternating between 30 min of milling and 10 min of rest. All manipulation of materials was conducted in an argon‐filled glove box with H_2_O and O_2_ levels below 1 ppm to prevent contamination by air.

*Characterizations*: Thermogravimetric analysis (TG; Netzsch STA 449 F3) was conducted on equipment connected to a mass spectrometer (MS; Hidden HPR 20) using a heating rate of 5 °C min^−1^ under dynamic argon with a purge rate of 80 mL min^−1^. The phase composition of the powdery composite was analyzed by X‐ray diffraction (XRD, D8 Advance, Bruker AXS) with Cu Kα radiation. To prevent any possible reactions between the sample and air during the XRD measurement, amorphous tape was used to cover the samples. Fourier transform infrared (FTIR, Magna‐IR 550 II, Nicolet) analysis was adopted to verify the chemical bonding. During FTIR measurements (KBr pellets), a closed tube was used to load samples with KBr for measurement in an argon‐filled glove box. The morphology of the samples was determined using a field emission scanning electron microscope (FE‐SEM, JEOL 7500FA, Tokyo, Japan) and a transmission electron microscope (TEM, JEOL 2011 F, Tokyo, Japan). Elemental analysis was performed with an Elemen Tar Vario EL3 Elemental Analyser. The XPS were obtained on a Perkin Elmer PHI 5000C ESCA system equipped with a dual X‐ray source, in which an Mg Kα (1253.6 eV) anode and a hemispherical energy analyzer were used. The background pressure during data acquisition was maintained at <10^−6^ Pa, and measurements were performed at a pass energy of 93.90 eV. All binding energies were calibrated using contaminant carbon (C 1s = 284.6 eV).

The hydrogen storage properties of the thus‐synthesized hydrogen storage materials were investigated on a Sieverts apparatus, denoted as a gas reaction controller (GRC, Advanced Materials Corp., USA). Rehydrogenation of LiBH_4_@G was conducted at 320 °C under an initial H_2_ pressure of 100 atm for 5 h, and the desorption properties were determined at various temperatures under a hydrogen pressure below 0.01 atm.

## Supporting information

Supplementary
