# Supplementary material for: Oxygen‐free Layer‐by‐Layer Assembly of Lithiated Composites on Graphene for Advanced Hydrogen Storage
Source: Adv Sci (Weinh). 2017 Apr 25;4(9):1600257. doi: 10.1002/advs.201600257 (PMC5604367; doi:10.1002/advs.201600257)
Supplement: Supplementary file 1 — Supplementary [file ADVS-4-na-s001.pdf]

Copyright WILEY-VCH Verlag GmbH & Co. KGaA, 69469 Weinheim, Germany, 2016.

ADVANCED SCIENCE
Open Access

# Supporting Information

for *Adv. Sci.,* DOI: 10.1002/advs.201600257

## Oxygen-free Layer-by-Layer Assembly of Lithiated Composites on Graphene for Advanced Hydrogen Storage

*Guanglin Xia, Yingbin Tan, Xiaowei Chen, Fang Fang, Dalin Sun, Xingguo Li, Zaiping Guo,* and Xuebin Yu**

Copyright WILEY-VCH Verlag GmbH & Co. KGaA, 69469 Weinheim, Germany, 2013.

# Supporting Information

**Oxygen-free Layer-by-Layer Assembly of Lithiated Composites on Graphene for Advanced Hydrogen Storage**

*Guanglin Xia, Yingbin Tan, Xiaowei Chen, Fang Fang, Dalin Sun, Xingguo Li, Zaiping Guo,* and Xuebin Yu**

## Methods:

### 1. Calculation:

Total energy calculations were conducted based on density functional theory (DFT) implemented in the Vienna *ab-initio* simulation package (VASP),[S1] using the generalized gradient approximation of Perdew-Burke-Ernzerhof.[S2,S3] The electron-ion interactions were calculated by the projector-augmented wave approach.[S4] In order to ensure sufficient convergence (less than 1 meV/cell), the energy cut-off for the plane wave expansion was set to 400 eV. The Brillouin zones were sampled by Monkhorst-Pack k-point meshes[S5] for all compounds, with meshes chosen to give a roughly constant density of k points (20 $Å^3$) for all compounds. Tests showed that our choice of k points yielded energies that converged within 0.01 eV/(f.u.). Graphene sheet was modeled using a 40 × 25 × 22 Å supercell containing 108 C atoms of graphene plus 28 H atoms saturating the dangling bonds at the edges. In this case, the graphene atoms on adjacent nanostructures are separated by over 10 Å in the x and y directions, and 22 Å in the z direction.

To understand the interaction between $C_4H_9Li$ and hexane and graphene, the adsorption of $C_4H_9Li$ and $C_6H_{12}$ on the graphene was studied, respectively. Usually two subsystems ($C_4H_9Li$ in hexane and graphene) interact with each other through the van der Waals interactions. Nonetheless, this interaction cannot be precisely described by either the local density approximation (LDA) or the generalized gradient approximation (GGA) functional. Therefore, the DFT-D2 method which adds a semi-empirical pairwise force field to

conventional DFT calculations was used to estimate the binding strength. The spacing between graphene and $C_4H_9Li$ or $C_6H_{12}$ was fixed, while their planar coordinates were fully relaxed to calculate the binding energy. Figure 3 displays the adsorption configuration and calculated binding energy as a function of the molecule-sheet separation. The binding energy curves suggest a binding ground state between $(C_4H_9)_2Mg$ ($C_4H_9Li$) and graphene, with an equilibrium distance of 2.43 Å (2.57 Å) and binding energy of -0.699 eV (-0.616 eV), while the $C_6H_{12}$-graphene system shows an equilibrium distance of 2.52 Å and binding energy of -0.309 eV.

The binding energy of the $C_4H_9Li$-graphene system is significantly lower than that of the $C_6H_{12}$-graphene system, indicating that the interaction between $C_4H_9Li$ and graphene is very much stronger than that of the $C_6H_{12}$-graphene system. Therefore, in contrast to $C_6H_{12}$, $C_4H_9Li$ can be easily attached on graphene.

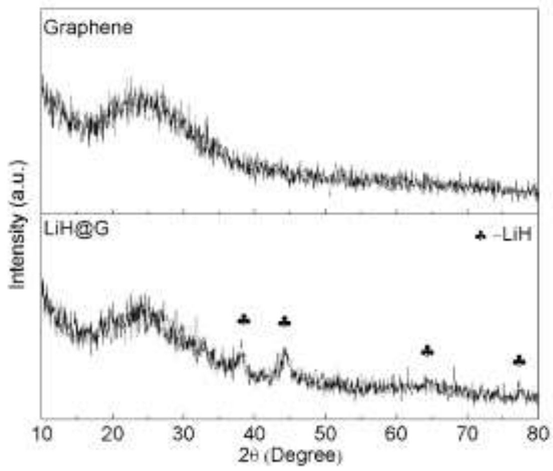


Figure S1. XRD patterns of the pristine graphene and the as-synthesized LiH@G.

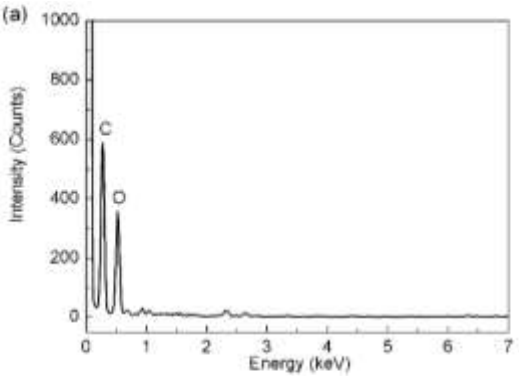


Figure S2. EDX spectrum of the as-prepared LiH@G.

Only signals of C and O belonging to GR were detected in the spectrum of LiH@G, indicating that the resulting composite was composed of light elements ($Z \leq 5$), viz., LiH.

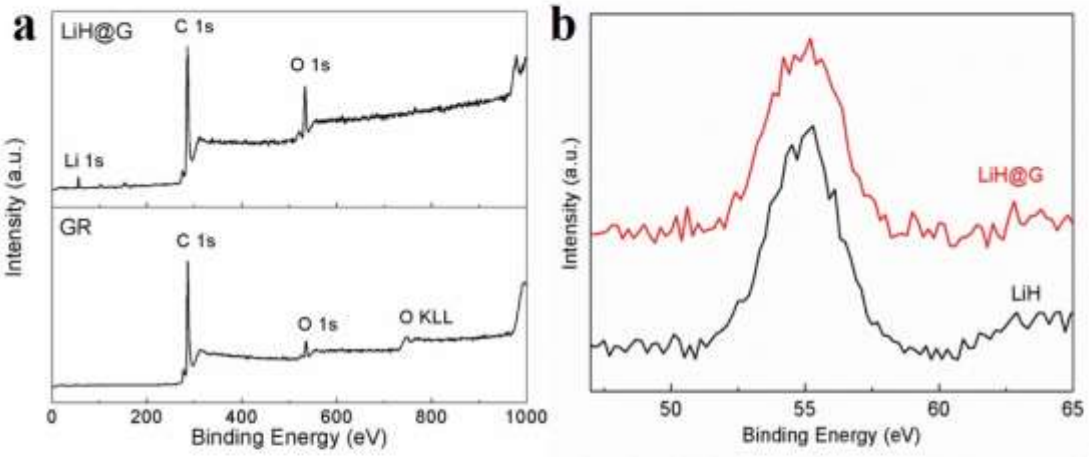


Figure S3. (a) XPS spectra of the LiH@G (top) and graphene (bottom). High-resolution Li 1s spectra (b) of LiH@G in comparison with pure LiH.

The Li 1s peaks in LiH and LiH@G show a single component due to the contribution of the hydride (LiH) and the associated oxide ($Li_2O$), which is formed during the measurement after contact with air.

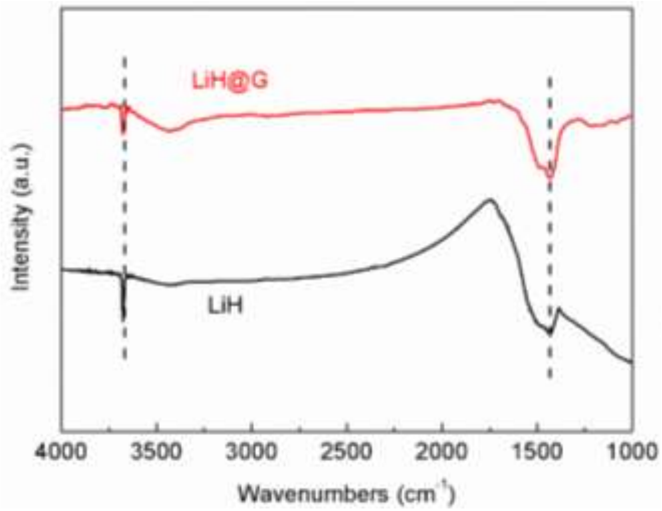


Figure S4. FTIR spectra of the as-synthesized LiH@G in comparison with LiH, which shows the characteristic peaks belonging to LiH. It indicates the successful formation of LiH in LiH@G.

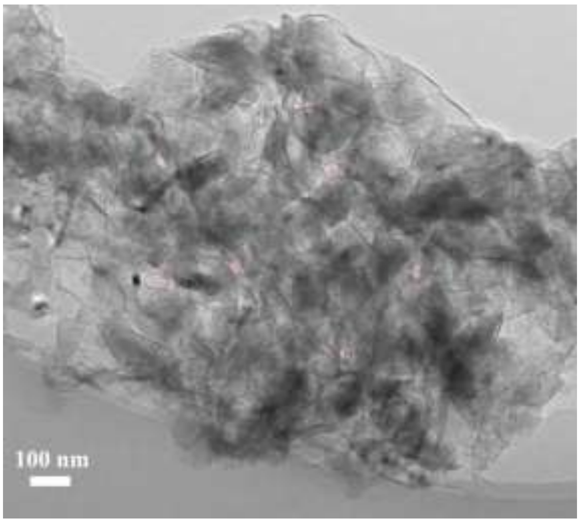


Figure S5. TEM image of the as-synthesized LiH@G. The red circle clearly shows the nanopores resulting from the stacking and cross-linking of LiH nanosheets and flexible graphene nanosheets.

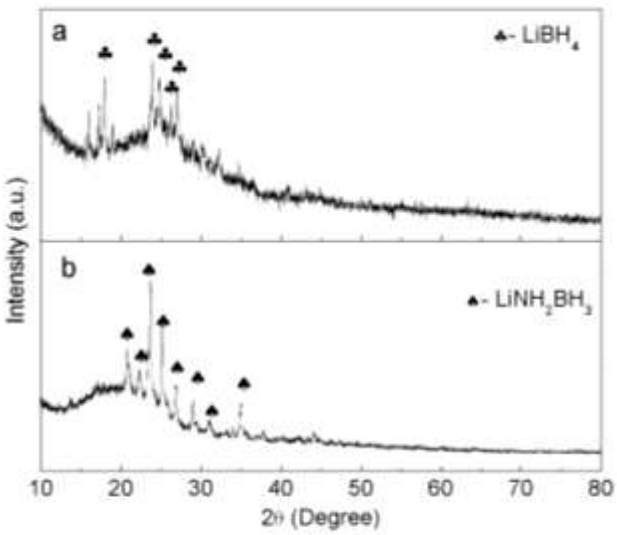

Figure S6. XRD patterns of the resulting $LiBH_4$@G (a) and $LiNH_2BH_3$@G (b).

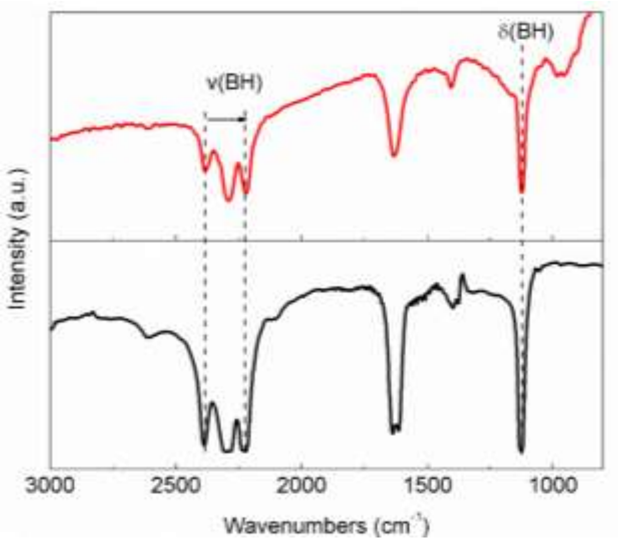


Figure S7. FTIR spectra of the as-synthesized $LiBH_4$@G (red line) in comparison with bulk $LiBH_4$ (black line).

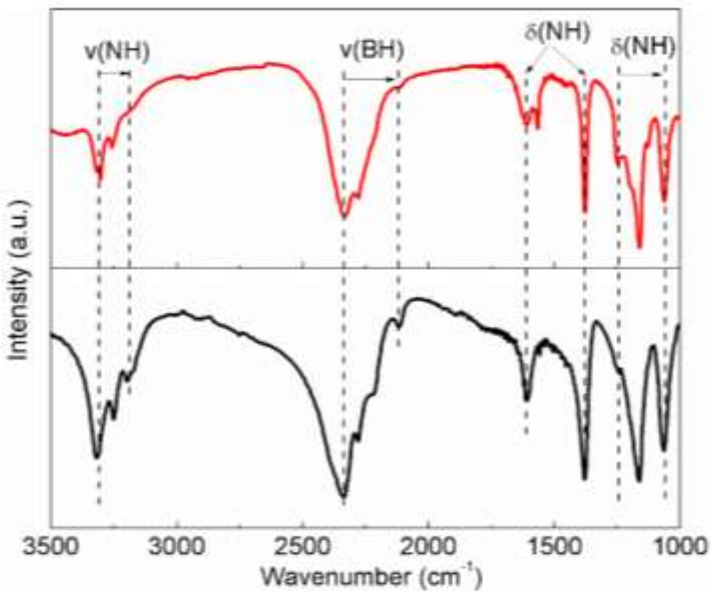

Figure S8. FTIR spectra of the as-synthesized $LiNH_2BH_3$@G (red line) in comparison with bulk $LiNH_2BH_3$ (black line).

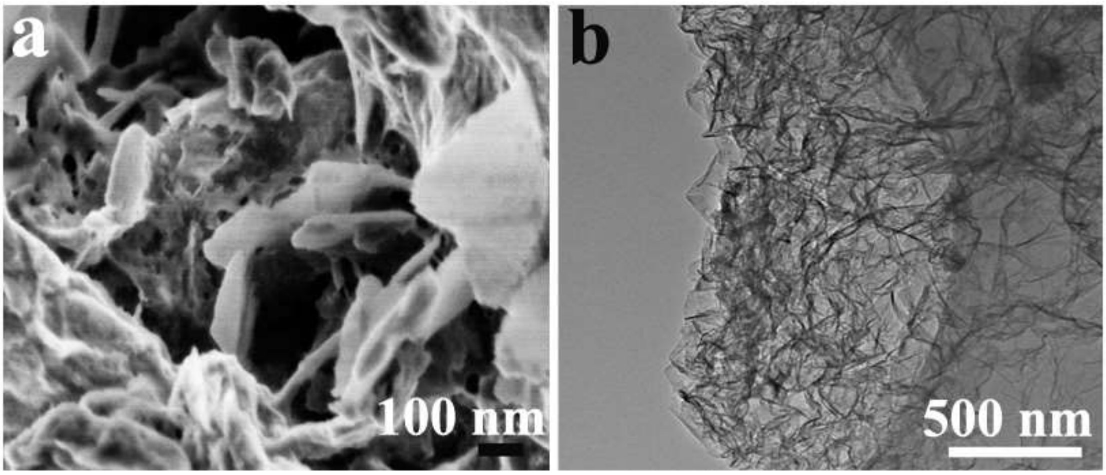


Figure S9. SEM (a) and TEM (b) images of the as-synthesized $LiNH_2BH_3$@G.

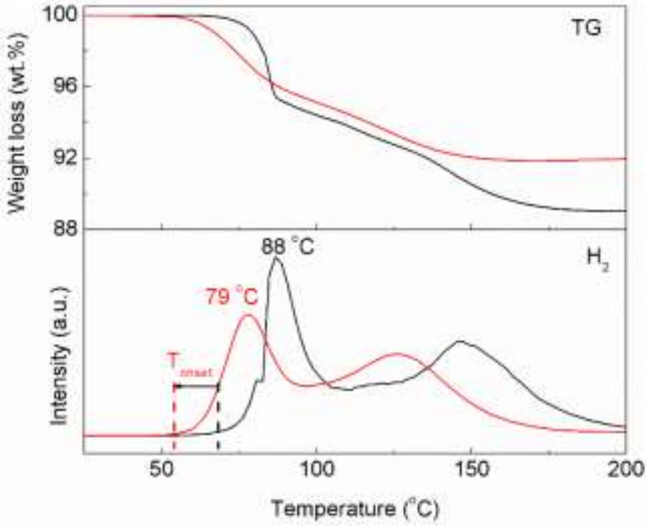


Figure S10. TG (top) and MS (bottom) results for $LiNH_2BH_3$@G (red line) in comparison with bulk $LiNH_2BH_3$ (black line).

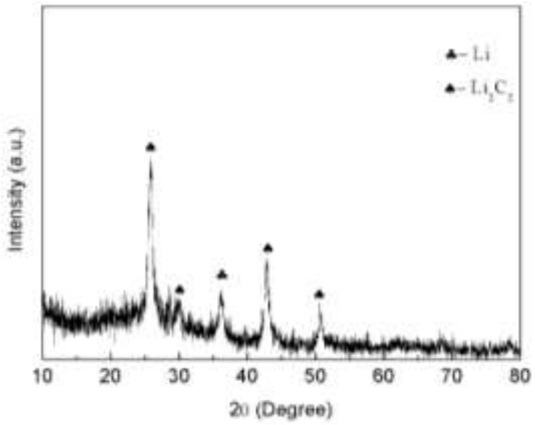


Figure S11. XRD patterns of the dehydrogenated products of $LiBH_4$@G after heating to 550 °C. The disappearance of $LiBH_4$ and LiH coupled with the presence of Li and $Li_2C_2$ demonstrates the complete decomposition of $LiBH_4$ after heating to 550 °C.

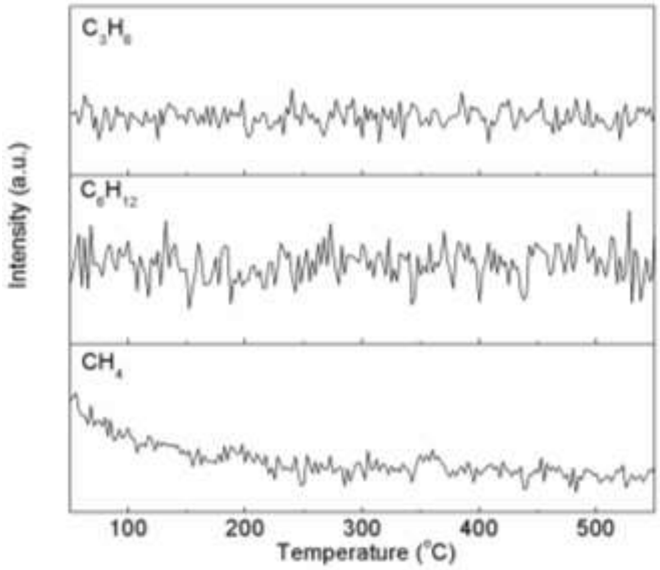


Figure S12. MS signals of possible byproducts from $LiBH_4$@G upon heating to 550 $^{o}$C.

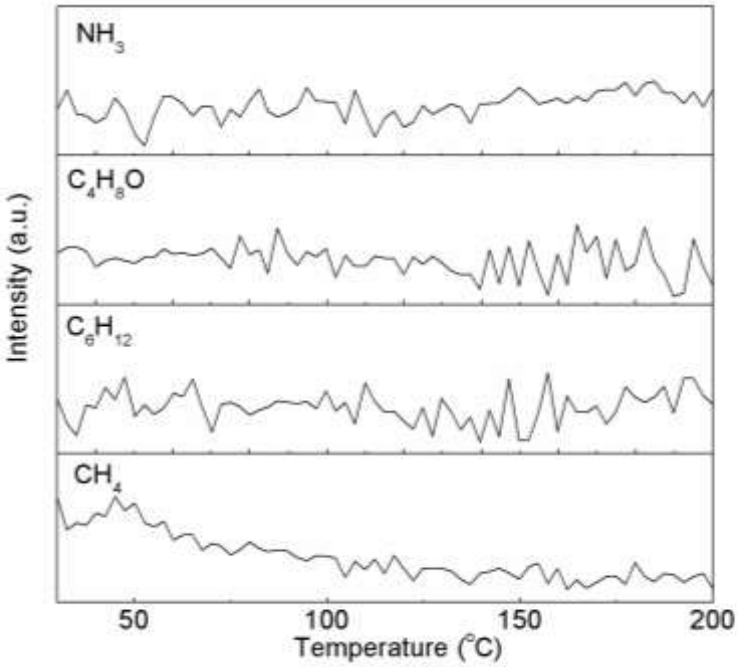


Figure S13. MS signals of possible byproducts from $LiNH_2BH_3$@G upon heating to 200 °C.

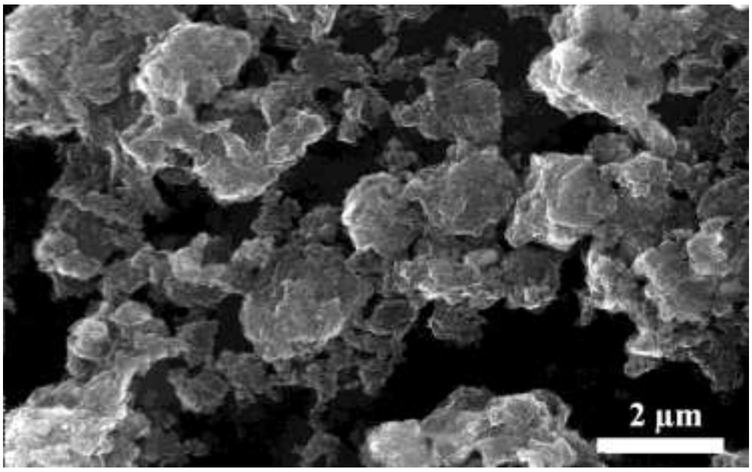


Figure S14. SEM image of the ball-milled composite of $LiBH_4$/G.

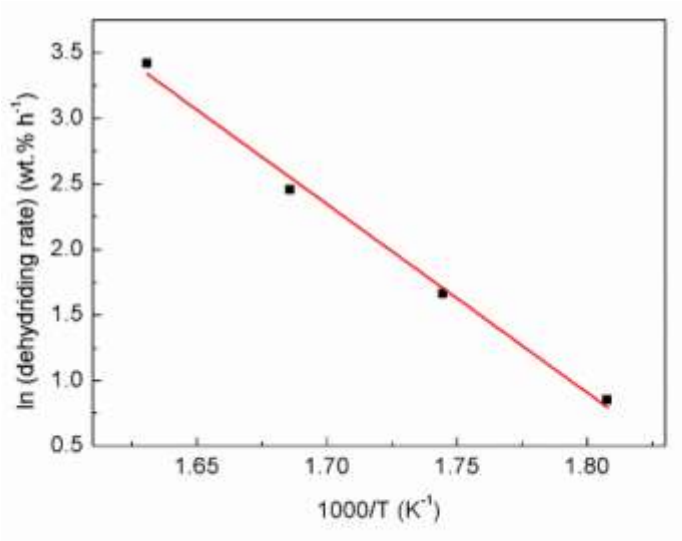


Figure S15. Arrhenius profiles of the dehydrogenation kinetics of the $LiBH_4$@G.

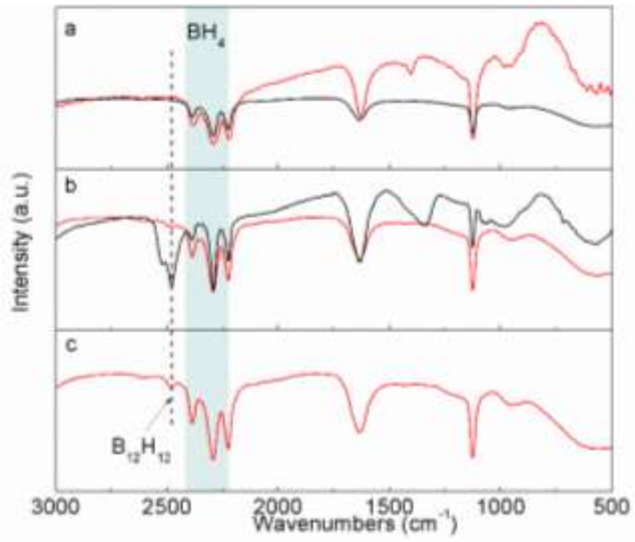


Figure S16. FTIR spectra of (a) the as-synthesized $LiBH_4$@G (red line) in comparison with bulk $LiBH_4$ (black line), and their products after the 2nd (b) and the 4th (c) cycles of hydrogenation.

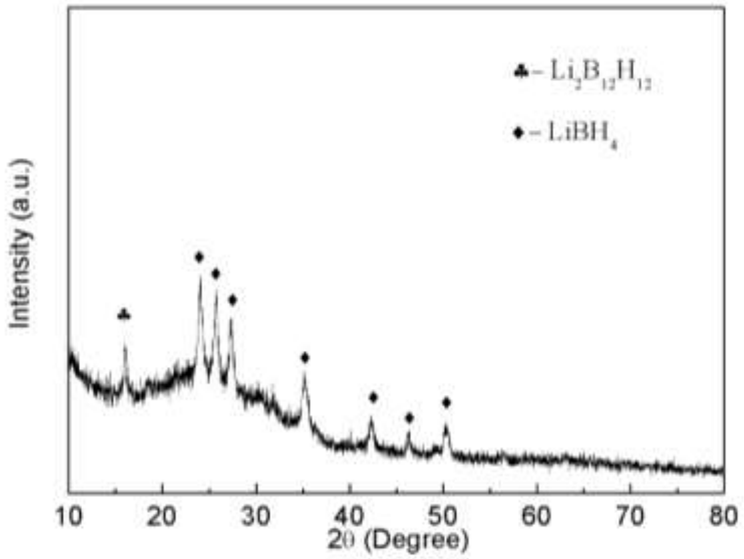


Figure S17. XRD patterns of $LiBH_4$@G after 4$^{th}$ cycle of hydrogenation.

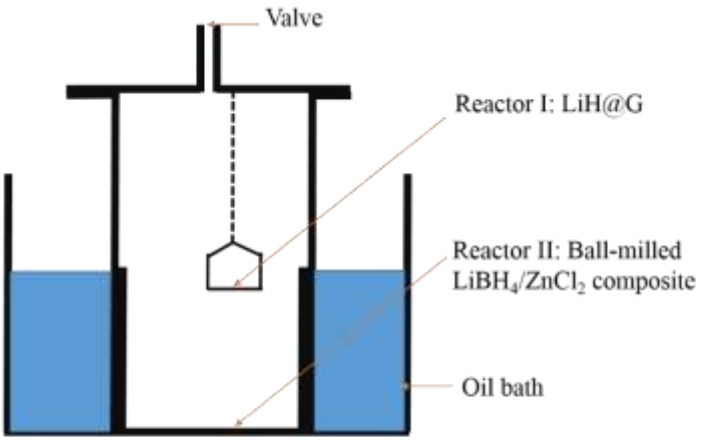


Figure S18. A schematic diagram of the preparation of $LiBH_4$@G from LiH@G.

## References:

S1. Kresse G. and Furthmüller J. *Phys. Rev. B*, **1996**, 54, 169.
S2. Perdew J. P., Burke K. and Ernzerhof M. *Phys. Rev. Lett.*, **1996**, 77, 3865-3868.
S3. Perdew J. P., Burke K. and Wang Y. *Phys. Rev. B*, **1996**, 54, 16533-16539.
S4. Blöchl P. E., Projector augmented-wave method, *Phys. Rev. B*, **1994**, 50, 17953.
S5. Monkhorst H. J. and Pack J. D. *Phys. Rev. B*, **1976**, 13, 5188-5192.
